# A Functional Review of Research on Clarity, Immediacy, and Credibility of Teachers and Their Impacts on Motivation and Engagement of Students

**DOI:** 10.3389/fpsyg.2021.712419

**Published:** 2021-06-30

**Authors:** Jin Zheng

**Affiliations:** School of International Education, Yellow River Conservancy Technical Institute, Kaifeng, China

**Keywords:** teacher's interpersonal communication, positive psychology, teacher's clarity, teacher's credibility, teacher's immediacy, motivation, engagement

## Abstract

The interpersonal communication behaviors of teachers have been substantiated to affect motivation, engagement, and success of students in the academic arena. Aiming to provide a systematic review of some teacher-related constructs in this domain, the present article was a bid to explain the crucial pillars of clarity, credibility, and immediacy of teachers in juxtaposition with theories and models of motivation and engagement of students. More particularly, this article presents some theoretical underpinnings to justify its claims using the ideas of positive psychology, the broaden-and-build theory, the rhetorical/relational goal theory, social cognitive theory, the attachment theory, some popular motivational theories, and the engagement theory. These theories signify the importance of clarity, credibility, and immediacy of teachers in the classroom and depict their association and impact on motivation and engagement of students. Later, the arguments are defended through a quick glance at the available empirical studies on each of the constructs. Afterward, the findings and implications of this review article are discussed. Finally, research gaps and future directions are presented for avid researchers to make new explorations.

## Introduction

Undoubtedly, teachers are one of the most important stakeholders in all educational milieus who can vastly determine the rate and quality of achievement and communication ability of students, especially in an English as a foreign language (EFL) context, in which students largely rely on their teachers for their learning and development (Pishghadam et al., 2019, 2021). Hence, the classroom decisions that teachers make put a huge impact on the achievement and perception of students (Danielson, 2002). Along with the decisions and actions of teachers, their behavioral, psychological, and instructional features are prominent in academia and L2 education (Burroughs et al., 2019; Derakhshan et al., 2020). Obviously, in order to make learning occur and to help students experience wellbeing, teachers must take emotions of their learners into account (MacIntyre et al., 2019). This conceptualization emanated from positive psychology (PP), which explores how people thrive and flourish *via* the strengths and virtues that make life good (Csikszentmihalyi and Nakamura, 2011). PP is said to be a rebirth for the humanistic psychology, which has a short history yet a long past (Macintyre and Mercer, 2014). It differs from humanistic psychology in that PP gives more weight to empirical research (Macintyre and Mercer, 2014). This theory rests on three pillars, namely, *positive emotions, positive character traits*, and *positive institutions* (Seligman, 2011). Along the same line, in his seminal work on PP and wellbeing, Seligman (2011) proposed a model which was built of five elements, namely, positive emotion, engagement, positive relationships, meaning, and accomplishment (PERMA). These studies, rightly, signify the importance of emotions and psychological factors of learners in the processes of learning and teaching.

Moreover, as pinpointed by Habash (2010), to improve the teaching quality, teachers should employ effective strategies to capture the interests of learners and engage them in interactive activities in the classroom. One of the venues for reflecting care of teachers for comprehension and gain in learners is having clarity in the class. Teacher clarity is of significant value in interactive contexts, in which the teacher and the learner mutually affect the learning and teaching processes. By definition, teacher clarity refers to the strategies and approaches that teachers employ to make sure that students have mastered the course content and processes (Bolkan, 2017). Having positioned itself in the body of knowledge in this area, teacher clarity has been in the limelight over the past decade through a proliferation of research. The results of numerous studies certify that it significantly improves cognitive learning, has a strong impact on affective learning, promotes student success, and prompts positive emotional responses that direct students to higher levels of engagement and motivation (Titsworth et al., 2015; Bolkan, 2017). Similarly, in their recent investigation, Roksa et al. (2017) maintained that student motivation, engagement, and faculty interest account for two-thirds of the correlation between teacher clarity and academic success. These studies substantiate that, in the context of English Language Teaching (ELT), which is now interaction-oriented in many countries with the rise of the communicative language trend, both teachers and learners dynamically shape and reshape the process of learning *via* an iterative and negotiated instruction (Bolkan, 2017; Roksa et al., 2017). As a result, teachers must have clarity in order to bring about the final outcome, which is student learning. This clarity can represent itself in the organization, explanation, examples and practices, and assessment of teachers.

One of the most critical offshoots of teacher clarity is classroom immediacy as high clarity leads to high comprehension and achievement and this, in turn, establishes a positive and friendly rapport between the teacher and his/her students. Teacher immediacy is defined as a set of verbal and nonverbal behaviors and strategies that teachers use to create a sense of closeness with students (Cakir, 2015). The term was first proposed by Mehrabian (1969) in his landmark study on communication behaviors to denote the degree of closeness and rapport between people (Finn and Schrodt, 2012). As put by McCroskey and Richmond (1992), immediacy can manifest itself *via* verbal and non-verbal cues expressed by both students and teachers in the classroom in an attempt to build psychological and physical proximity between them (Wilson and Ryan, 2013; Delos Reyes and Torio, 2020). Furthermore, research suggests that teacher clarity and immediacy are two interrelated constructs, yet they function independently (Comadena et al., 2007; Titsworth et al., 2015). They are at variance in that teacher clarity arouses cognitive interest, but teacher immediacy arouses emotional interest (Mazer, 2013). Nevertheless, they both buttress student learning and achievement, as suggested by the *additivity hypothesis*, which puts forward that the two constructs join to form an ideal learning context for the learners (Titsworth et al., 2015). Like other strands of research, teacher immediacy has been scrutinized by different scholars in different educational contexts. The summation of the results of such studies point to the power of teacher immediacy to improve student empowerment and engagement (Cakir, 2015), reduce anxiety (Kelly et al., 2015), sustain student attention (Bolkan et al., 2017), and affect students in online learning contexts (Brooks and Young, 2015).

Besides its multiple benefits mentioned, teacher immediacy has a robust and positive relationship with teacher credibility, which is believed to be one of the most significant teacher-related variables in education (Teven and Hanson, 2004; Santilli et al., 2011). By essence, the notion of teacher credibility refers to the attitude of a learner toward his/her teacher, considering the competence, caring, and trustworthiness of the teacher (McCroskey and Young, 1981). Credibility has a significant role in determining the existing rapport in the classroom as learners hardly admit information from an incredible source. Moreover, the process of transmitting knowledge to students is not solely contingent upon the message and its presentation but partly on the degree of credibility in the source of the message (Teven, 2007). In addition to that, scientific findings indicate that teacher credibility improves student motivation and different aspects of learning (Johnson and Miller, 2002; Teven, 2007). Similarly, credibility has been identified to encourage students to learn affectively and cognitively (Pogue and AhYun, 2006). Perusing the available literature on these important variables (i.e., teacher clarity, immediacy, and credibility), one can find numerous research studies, each focusing on a specific aspect. However, to the best of the knowledge of the researcher, the conduction of a systematic review study on such factors in L2 education and their juxtaposition with one another have been largely ignored. Urged by this backdrop, the present study made an effort to systematically review the history, definitions, models, areas of research, gaps, and future directions concerning clarity, immediacy, and credibility of teachers and their impacts on motivation and classroom engagement of students.

## Theoretical Foundations

### Positive Psychology

For a long time, applied linguistics has been ruled by cognitive perspectives, and this has ended in an underestimation of the crucial role that emotions play in foreign language learning and teaching (Dewaele et al., 2019). Nevertheless, in the past couple of decades, the false idea that exploring the emotions of an individual is in some way unscientific has been superseded by a drastic shift branded as “emotionology” after a groundbreaking study conducted by Mackenzie and Alba-Juez (2019). Such a trend grew like a snowdrift by the passage of time, and emotional aspects of language learning and teaching boomed unprecedentedly among scholars (Prior, 2019). Now, “pigs can fly” and the centrality of affect and emotion has witnessed a bulk of research after the emergence of a new field called “positive psychology.” PP focuses on the exploration of how people thrive and flourish (Macintyre and Mercer, 2014) and capitalizes more on the positive side of life as opposed to the exclusive focus of general psychology on problems and abnormalities (Seligman, 2006). The central aim of this school of psychology is to assist people to live a better life and to create instruments to constitute positive emotions, to upsurge engagement, and to make life meaningful (Seligman, 2006).

Positive psychology takes a giant step and goes beyond the investigation of “anxiety, motivation, and attitude.” Instead, it focuses on the role of constructs and attributes such as courage, perseverance, wellbeing, flow, optimism, hope, resilience, creativity, and happiness. As for its components, Seligman and Csikszentmihalyi (2000) contended that PP is set up on three core pillars, namely, “positive subjective experience, positive individual traits, and positive institutions” (p. 6). Positive subjective experience concerns internal experiences, such as emotions; positive individual traits refer to traits associated with living well; and positive institutions are the institutional contexts that allow people to develop (Macintyre and Mercer, 2014). Spreading the seeds on PP on the ground of applied linguistics, scholars carried out numerous investigations on the role of emotions and attitudes of stakeholders in incurring learning in different educational settings. This development blossomed with the burgeoning body of research on emotions, affective factors, and positive affects in L2 education (Dewaele, 2015). However, this focus on emotional dimensions in applied linguistics has been biased toward negative emotions, and PP is still in the shadow of cognitive-oriented outlooks (Dewaele et al., 2019).

Although PP and its tenets bring about many benefits for L2 learners and teachers, such as improving their resiliency and hardiness to cope with future negative events, taking into account their affect, and moderating the influence of negative emotions (Gregersen, 2013; Dewaele et al., 2019), this science has also been criticized for its exhaustive employment of cross-sectional designs, simplistic treatment of positive and negative emotions, ignorance of individual and group differences, and, finally, weak measurement of emotions (Lazarus, 2003). Yet, researching the unexplored avenues of this scholarly domain and resolving its problems is a tough nut to crack in many educational contexts, and the present review aims to propose some directions in this regard.

### The Broaden-And-Build Theory

As one of the offshoots of PP, the broaden-and-build theory proposed by Fredrickson (2001) pays special attention to positive emotions in flourishing and continued actions of humans. It makes a distinction between positive and negative emotions. Based on this theory, positive emotions (e.g., joy, interest, and love) have five core functions: (1) they broaden thought-action repertoires, (2) undo the enduring impacts of negative emotions, (3) improve psychological resiliency, (4) build personal resources, and (5) produce psychological and physical wellbeing. On the other hand, negative emotions, such as anxiety, tension, and fear, have detrimental effects on people as they narrow thought–action repertoires of an individual and limit his/her level of performance (Macintyre and Mercer, 2014).

The logic behind this theory is the conception that positive emotions can cause ideal short-term and long-term functioning. Consequently, these emotions are of paramount importance, which need to be cultivated in individuals not only as an end but also as a means to promote psychological and physical wellbeing (Fredrickson, 2004). In the context of L2 education, positive emotions are significant as a learning structure that cares for influential factors, inspires student perseverance and pursuits of thoughts and actions, and facilitates the ground for optimal motivation, learner engagement, and learning (Rahimi and Bigdeli, 2014). Another benefit of positive emotions is that they help teachers to eradicate and regulate negative emotions, such as stress, anxiety, and fear, and even alter them to positive emotions (Fredrickson, 2004). Hence, L2 teachers and material developers are expected to utilize techniques, strategies, and tasks that reflect their care for augmenting positive emotions and preventing negative emotions in an attempt to establish a learning atmosphere in which students can flourish and become tough and resilient metals which “bend but not break.”

### The Rhetorical/Relational Goal Theory

The rhetorical/relational goal theory (RRGT) is a theory in the realm of instructional communication, which was proposed by Mottet et al. (2006) to see how the process of instructional communication operates. Based on this theory, both teachers and students have rhetorical and relational goals in the classroom that they wish to attain (Mottet et al., 2006). Therefore, each classroom centers around the needs/goals of learners and instructors who have relational needs (e.g., to be liked and admitted) and rhetorical needs (e.g., to accomplish a task and to gain a specific grade). The crucial responsibility is put upon shoulders of teachers as they are expected to manage both relational and rhetorical needs at the same time through their behavioral choices to fulfill the classroom needs; and once these goals are obtained and student needs are satisfied, then the optimal learning can take place (Frymier, 2007). It is worth noting that the rhetorical and relational behaviors of teachers serve different purposes. For instance, teachers employ rhetorical instructional communication behaviors (e.g., clarity) to promote effective teaching and affect beliefs, attitudes, and behaviors of learners in the class *via* shaping their designed instructional messages (Beebe and Mottet, 2009). On the other hand, teachers use relational instructional communication behaviors (e.g., nonverbal immediacy) to stir the formation of a mutually shaped professional relationship and rapport with their students (Myers, 2008).

As in other areas of education, teachers and students have different ways of forming rhetorical and relational goals. They both have academic and relational needs, which need to be attended to in academia in order to provide a friendly learning context that fosters motivation and optimum achievement among students. In sum, as research shows, in any learning context, teachers must use a blend of rhetorical and relational behaviors in order to bring about favorable outcomes (Myers et al., 2018). Such instructional behaviors of teachers may be affected by other related factors, such as the teaching context, personality of teachers, perceptions of students, and credibility, clarity, and homophily of teachers (Dunleavy, 2006; Beebe and Mottet, 2009).

### Social Cognitive Theory

Social cognitive theory (SCT) is a learning theory, proposed by Bandura (1986), which asserts that learning happens in a social environment with a dynamic and mutual interaction among the individual, context, and behavior (also known as *reciprocal determinism*). SCT highlights the crucial role of social influence and external/internal social support as its core features. SCT considers cognitive aspects of behavior and learning, in which the person obtains and preserves behavior and at the same time confirms the power of social setting wherein people carry out the behavior. As pinpointed by Bandura (2008), SCT posits that, when individuals observe behavior of another individual (a model) and its consequences, they recall this sequence in their subsequent behaviors. In other words, SCT underscores the prominence of behavioral observation, modeling, and imitation and the effect of attitudes and emotional reactions of others in behavior and learning of an individual (Bandura, 1986). Therefore, SCT deals with how environmental and cognitive factors interact to impact the learning and behavior of people. This theory has long been associated with a set of important constructs of learning and development, namely, reciprocal determinism, behavioral capability, observational learning, reinforcement, expectations, and self-efficacy.

Despite its benefits and potentials, SCT has been criticized for its overestimation of the role of the environment in determining behavior of an individual, weak organization and basis, the unclear idea of the amount of power among each of the factors (person, context, and behavior), negligence of maturation and biological–hormonal tendencies that can affect behaviors of an individual, overemphasis on emotion and motivation in relation to past experiences, and difficult application (see Schunk, 2012).

### The Attachment Theory

The attachment theory (AT) was proposed by Bowlby (1969) to describe relational patterns among people. It has been regarded as one of the cornerstones of developmental psychology and child maturation, which posits that attachment of a child to a caregiver generates a type of behavior that can later become autonomous. Although this theory has been mostly associated with the development of a child and romantic relationships of adults, it has also been applied in the context of language education by some scholars, such as Geddes (2006) and Fleming (2008). Attachment is an emotional tie among individuals that can affect their relationships, experiences, and engagement in activities. In the context of L2 education, it can be claimed that students develop emotional attachments with their teachers and peers. Such attachments can be secure or insecure. According to AT, those students who form an attachment with their teacher are relaxed to make explorations, and such emotional attachments can become the foundation of their socialization (Bergin and Bergin, 2009). Another benefit of student–teacher attachments is creating risk-taking learners who are not afraid of failure and are highly motivated and engaged in classroom activities (Bergin and Bergin, 2009). In such a stress-free and friendly environment, the immediacy and rapport of teachers with their students is high, and in case a learner does not know a concept, he/she is not flushed and stressed out. In sum, teachers must establish secure attachments with their students in order to provide them with a ground for their progress and success. Correspondingly, the rapport and immediacy of teachers will increase in such an environment.

### Motivation and Engagement of Students: Theories and Models

The construct of motivation has long been regarded as the core of success and achievement of humans in both personal and academic life (Gopalan et al., 2017). It is the spirit behind any action that a person takes without which nothing is possible. In educational domains, motivation has been numerously investigated from different angles and perspectives, which unanimously affirmed the impact of this construct on the achievement and learning of learners (see Gardner, 2010; Dörnyei and Ushioda, 2011; Al-Hoorie, 2017). In a period of around 64 years of motivation research, different theories and models have been proposed by different scholars and leading figures of this strand of research, which is still developing. Among those models, Intrinsic and Extrinsic Motivation Theory, Self-Determination Theory (SDT), The ARCS Model, SCT (explained earlier), and Expectancy Theory have been the most widely cited and employed ones in the pertinent literature. Intrinsic and Extrinsic Motivation Theory considers motivation to be driven by internal and external forces. When a person does an activity only for self-satisfaction and enjoyment, his/her motivation is said to be intrinsically formed. On the other hand, when the motivation and drive of an individual to complete a task is to gain an external reward or appraisal, he/she is believed to be extrinsically motivated (Brown and Lee, 2015). According to this theory, both types of motivations have positive and negative points. Yet, intrinsic motivation is more effective in the complex process of language learning as it inspires the interaction, effort, and long-run performance in the learner (Pinder, 2011).

Another popular theory in this domain is that of SDT established by Ryan and Deci (2000). It is a macro theory of personality and motivation whose fundamental focus is on inner motives of a human for development and change, together with their psychological needs. Additionally, SDT postulates that human beings can become self-determined in case three core needs of *competence, relatedness*, and *autonomy* are satisfied in them. The concept of self-determination means that one is able to control and manage his/her behavior and action. Given this, once a student considers him/herself as capable of controlling and managing a task, he/she would invest more time and energy in accomplishing the task properly. The key to the three core needs of this theory is the “sociocultural context” in which one carries out a task (Legault, 2017). The next seminal model in motivation research is the attention, relevance, confidence, and satisfaction (ARCS) model proposed by Keller (1983). It is a synthetic approach to motivation, which pinpoints four key components of learning that motivate the students and maintain their spirit: attention, relevance, confidence, and satisfaction. The model comprises different categories and subcategories of motivational attributes with the purpose of identifying motivational features of students and developing suitable remedial techniques and strategies to form and maintain their motivation. The final theory of motivation to be explained here is the Expectancy Theory, which was first introduced by Vroom (1964) and later developed by Van Eerde and Thierry (1996). The theory is based on the idea that an individual makes more attempts if he/she expects the outcome of the action to be valuable and rewarding. Like other theories, there are essential components in this theory as well. It has three core elements: valence, instrumentality, and expectancy (VIE). Valence is the importance that one places on something, instrumentality refers to the dependency of reward on the performance of an individual, and, finally, expectancy denotes the expectation of an outcome through increased effort (Vroom, 1964).

As for the last variable of concern in this review study (i.e., student engagement), there is a popular theory known as the Engagement Theory which capitalizes on the meaningful involvement of the students in their learning by means of interactive tasks (Miliszewska and Horwood, 2006). The theory was originally proposed by Kearsley and Shneiderman (1998) and has three main principles: “*Relate*,” or learning *via* communication and collaboration; “*Create*,” or learning through creative and project-based activity; and “*Donate*,” or learning through having an outside focus. This theory improves team learning, collaboration, and large community involvement (Kearsley and Shneiderman, 1998). This theory is related to motivation, in that an engaged student in the classroom will exert more effort when he/she finds the tasks meaningful and transferable to real life. In simple words, engaged students acquire and keep motivation more easily as opposed to demotivated students.

## Empirical Studies: A Cursory Glance

A bulk of research studies in the available literature has indicated that the positive emotions and interpersonal communication behaviors of teachers affect the learning process of students (Gabryś-Barker, 2016). The reason behind this mutual impact in the teaching profession is that positive student–teacher rapport is the basis of many successful classroom practices (Mercer and Gkonou, 2020; Strachan, 2020). Teaching is said to be a relational and negotiated job in which success is a function of many factors and stakeholders, not just the teacher. It is a myth to claim that students have narrow impacts on professional career of their teachers. Instead, even the identity of a teacher is co-constructed by the interactions that he/she has with his/her pupils and colleagues let alone other aspects (Beijaard et al., 2004). Many studies provided evidence for this mutual relationship and pinpointed that engagement, achievement, wellbeing, motivation, success, hope, and so forth of students are correlated with the interpersonal communication behaviors of teachers (Frymier et al., 2019; Derakhshan, 2021; Pishghadam et al., 2021).

**Figure 1:**
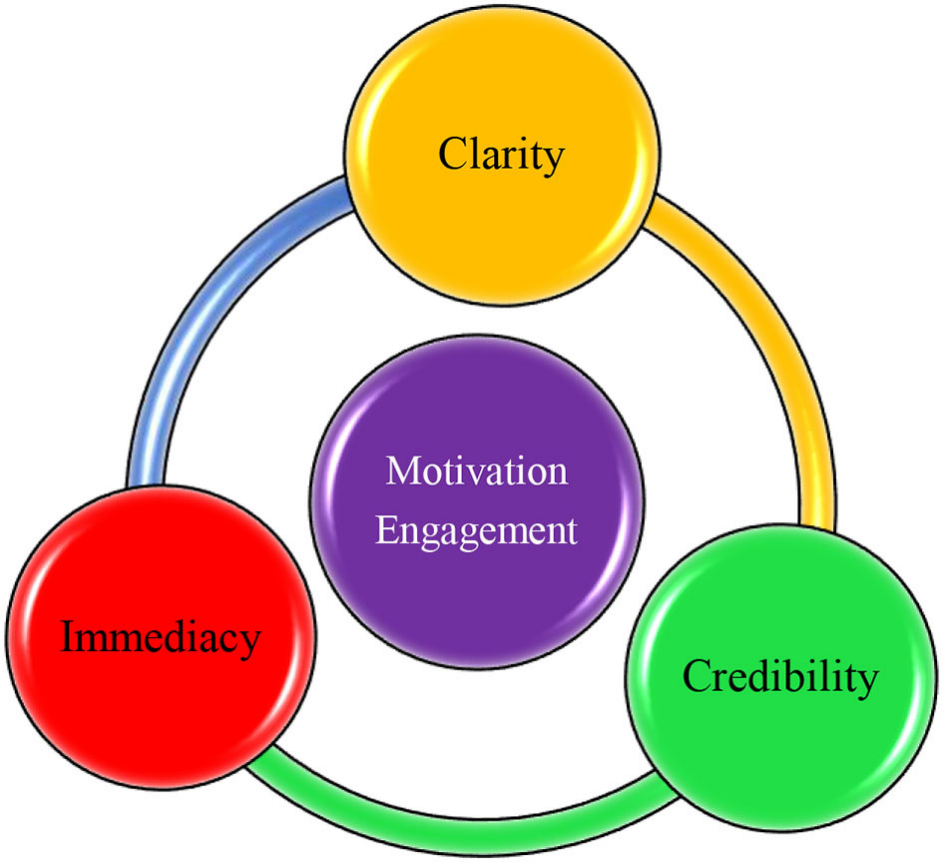
A visual model of the interpersonal communication behaviors of teacher and the outcomes of students.

One of the most important teacher-related constructs in this domain is teacher clarity (Figure 1), which has been defined as the strategies and approaches that teachers employ to make sure that students have mastered the course content and processes (Bolkan, 2017). It is a rhetorical behavior that signifies a process in which a teacher takes advantage of different verbal and nonverbal cues to effectively convey the meaning of course content to students (Myers et al., 2014). Existing literature on this variable affords a robust testimony for its positive impacts and associations with the cognitive learning of students (Bolkan, 2017), affect and affective learning (Mazer, 2013; Bolkan et al., 2016), motivation (Roksa et al., 2017), process, retrieval, and subsumption of information (Bolkan et al., 2016), deep learning (Blaich et al., 2016), academic success (Roksa et al., 2017), critical thinking (Wang et al., 2015), empowerment (Finn and Schrodt, 2012), and engagement (Roksa et al., 2017).

Another variable that was reviewed is teacher immediacy, which has been defined as verbal and nonverbal behaviors of teachers that decrease physical and psychological distance among individuals and constitute a sort of closeness (Mehrabian, 1967; Cakir, 2015). Such behaviors in the class are said to enlighten the classroom atmosphere and make students passionate and admitted by others (Li, 2003). Immediacy and liking are intertwined as they have a positive correlation with each other. When a teacher shows high immediacy, he/she is probably more liked by the students. Conversely, when the students like their teachers, this liking inspires the teacher to demonstrate more immediacy in the classroom (Mehrabian, 1971). This affiliation is a natural reaction in that we are prone toward people and things that we like and evade from those we dislike (LeFebvre and Allen, 2014). This emotional bond is of paramount significance in educational contexts as teachers are influential in the lives of students, and failure in this area will affect the ability of teachers to make an impact on the minds (almost impossible) of learners (Whitaker, 2006). The rich literature on teacher immediacy shows that it is a positive predictor of student learning (Violanti et al., 2018), reduced anxiety (Ballester, 2015), motivation (Frymier et al., 2019), sustained student attention (Bolkan et al., 2017), class attendance (Rocca, 2004), and engagement (Dixson et al., 2017; Derakhshan, 2021).

As pinpointed by Gray et al. (2011), a classroom is a persuasive context wherein the teacher as the authority of the class is in charge of convincing the students when facing opposition. This raises the importance of another interpersonal communication behavior (teacher credibility), which refers to the degree of competence, trustworthiness, and believability of a teacher from the perspective of the students (Teven and McCroskey, 1997). Based on this definition, in contrast with immediacy, teacher credibility is way beyond “liking” and concerns trust of students in the capability of the teacher to teach them something. In the classroom in which mostly teacher is the “influencer” and students are “influencee,” credibility is the basis of an effective influence as everything is contingent upon the credibility of the influencer (Hackman and Johnson, 2000). This line of research has been examined by different scholars from different parts of the world. Research indicates that this construct affects the motivation and learning of students (Johnson and Miller, 2002; Teven, 2007; Gray et al., 2011), affective and cognitive learning (Pogue and AhYun, 2006), class attendance (Pishghadam et al., 2021), and engagement (Derakhshan, 2021). Concerning the motivation and engagement of students, numerous studies have been conducted to explore and define the terms and propose comprehensive models (Dörnyei, 2009; Gardner, 2010; Dörnyei and Ushioda, 2011). However, due to space constraints and previously referred to studies throughout this review, the author avoids going through further studies and reinventing the wheel. The point is that both motivation and engagement have mostly been examined in relation to interpersonal communication behavior (e.g., credibility, care, immediacy) of a teacher in the pertinent literature rather than several behaviors, and this justifies the conduction of this theoretical review.

## Discussion

In this functional review, the author went through the definition and significance of positive emotions and interpersonal communication behaviors of teachers, making references to the theoretical foundations of this strand of research (i.e., positive psychology, the broaden-and-build theory, the rhetorical/relational goal theory, SCT, the attachment theory, motivational theories, and the engagement theory). Additionally, out of numerous communication behaviors, three constructs of teacher clarity, immediacy, and credibility were examined for their definitions, empirical studies, and their abundant effects on learning motivation and engagement of students. Besides, other areas of education, which are predicted by those three constructs, have been touched upon through presenting empirical studies. Based on this review, this research domain, which revolves around the positive emotions and rapport of teacher with students established by interpersonal communication behaviors, has many pedagogical implications for different parties in EFL contexts including students, teachers, teacher trainers, materials developers, supervisors, and researchers.

As for students, this strand of research can be of value in that it raises their awareness and knowledge of the fact that the teaching–learning process is a co-constructed phenomenon whose success and occurrence are not exclusively the duty of teachers. Students are equally influential in providing a fertile ground for their learning to occur. They should make attempts to establish a friendly relationship with their instructors, and this immediacy adds trust to the whole process of education thereby students flourish. When students are cognizant of their active role in (re)constructing professional identity of their teacher, they will show more motivation and engagement in their practices. In other words, the process of teaching–learning is like a nested system in which teachers and students have equal impact on one another. Teachers are the second group that can benefit from the ideas presented in this review article, in that they can get to know the importance of their interpersonal communication behaviors, especially their instructional clarity, immediacy, and credibility and their impacts on various aspects of education of students. Moreover, they can use this line of research to make reflections on their practices, identify the most appropriate strategies to establish a strong tie with their students, and facilitate the ground for their learning to occur. They can also develop their professional practice by working on their interpersonal/relational behaviors with students and meet their academic needs. Additionally, teachers can establish an approachable classroom rapport that stimulates academic success, alters behavior of students, and provides a conducive learning environment.

The next stakeholders to benefit from this study are teacher trainers, in the sense that they can cultivate in novice teachers the knowledge and application of interpersonal communication behaviors, in general, and teacher clarity, immediacy, and credibility, in particular. They can offer workshops, seminars, webinars, and other training courses in which pedagogical, psychological, relational, and affective aspects of teaching are equally taught. More specifically, teacher educators can afford teachers an opportunity to know the significance and use of interpersonal communication behaviors in the success of their profession. The propositions of this article are beneficial for material developers, in that they can design materials and textbooks in which, besides pedagogical concerns, interpersonal communication behaviors are also developed. They can write materials in such a way that teachers can work on their clarity, rapport with students, and credibility as teaching without believability works in vain. Similarly, supervisors can take advantage of this article to shift their attention in their supervisory practices from solely monitoring the pedagogical performance and practices of teachers to their interpersonal communication behaviors, without which their teaching is blind. The last group who can use the findings of this study consists of researchers who can conduct similar studies on other constructs of this line of research, such as teacher stroke, teacher care, rapport, confirmation, and humor using different instruments and designs in EFL, English as a second language (ESL), English for academic purposes (EAP), and English for specific purposes (ESP) settings.

In summary, the review of related literature on this strand of research shows a number of shortcomings that need to be resolved. First, despite the fact that interpersonal communication behaviors are equally important in the academic arena, only teacher immediacy has been sufficiently scrutinized (Dixson et al., 2017; Derakhshan, 2021), and other variables such as teacher care, confirmation, and stroke have been kept at the margins (Hsu, 2012; Pishghadam et al., 2021). This provides a good impetus for running analogous studies on the less researched variables of this area in relation to the demographic variables (age, gender, proficiency, etc.) to see if they still lead to academic success or not. Furthermore, future studies can examine teacher interpersonal communication behaviors from the perspective of the students to spot their perceptions, preferences, needs, and wants. Similar studies can also be conducted through online learning environments to explore if virtual communication mode makes a change in the mentioned constructs. Future scholars are recommended to investigate the sociocommunicative style of the teacher, and its effects on how interpersonal communication behaviors operate.

Tracing back the related literature on teacher clarity, immediacy, and credibility, one can easily notice that these important teacher-related variables have mostly been gauged *via* questionnaires and inventories (Finn and Schrodt, 2016; Pishghadam and Karami, 2017; Hall, 2019), and only in rare cases have qualitative measures like interview and case study been used (Barclay, 2012; Bondie and Zusho, 2017). The same story has happened in exploring the motivation and engagement of students (e.g., Appleton et al., 2006; Martin, 2008). All in all, what is missing in this scholarly territory is the use of qualitative-based tools to measure clarity, credibility, and immediacy of teachers, such as observation, portfolios, diaries, interventions, longitudinal studies, case studies, and interview panels with experts, all of which have many potentials to add to our interpretations of these constructs.

Future studies can be done on the process and development of these variables *via* longitudinal and case studies to identify the changes that teachers may undergo in the course of developing such communication behaviors. Similarly, portfolios, reflective journals, and think-aloud protocols can be used to see the mental processes that teachers and students experience in this regard. Treatments or experimental studies are also suggested to see if the instruction of such variables (e.g., clarity and credibility) is meaningfully different from those teachers who do not receive training. Another backdrop in this area of research is that culture, which has a considerable impact on all aspects of the life of an individual, has been scarcely investigated, and this is the Anglophone culture that has dominated this domain so far. Hence, intercultural and cross-cultural studies are recommended to examine the disparities that may exist between teachers of various cultural milieus despite the fact that some attempts have recently been made (e.g., Santilli et al., 2011; Derakhshan, 2021).

Moreover, the interpersonal communication behaviors of teachers, to date, have been mostly explored from the lens of the students, and the perspectives of other stakeholders have been overlooked. Future studies can work on the perceptions of supervisors, teacher trainers, and the teachers themselves. Narrowing their focus, avid researchers are suggested to investigate the impact of experience level, academic qualifications, age, and gender of teachers on their communication behaviors. Another problem of this scholarly domain is that it has mostly been scrutinized in the context of general education and hard sciences, and EFL/ESL environments with their specific interpersonal relations and dialogic nature (Mercer and Dörnyei, 2020) have captured insufficient attention among scholars. All these gaps signify that the research on this domain is still fresh with many unexplored territories for interested researchers. Therefore, scholars are universally invited to stand on the shoulders of the leading figures of this territory and run similar studies and ultimately add fresh insights to the body of knowledge in this area.

## Author Contributions

The author confirms being the sole contributor of this work and has approved it for publication.

## Conflict of Interest

The author declares that the research was conducted in the absence of any commercial or financial relationships that could be construed as a potential conflict of interest.
